# Comparisons of health-related quality of life among surgery and radiotherapy for localized prostate cancer: a systematic review and meta-analysis

**DOI:** 10.18632/oncotarget.21519

**Published:** 2017-10-05

**Authors:** Cheng Chen, Zhen Chen, Kun Wang, Linkun Hu, Renfang Xu, Xiaozhou He

**Affiliations:** ^1^ Department of Urology, The Third Affiliated Hospital of Soochow University, Changzhou, Jiangsu, P.R. China; ^2^ Department of Urology, The First Affiliated Hospital of Soochow University, Suzhou, Jiangsu, P.R. China

**Keywords:** prostatic neoplasms, prostatectomy, radiotherapy, quality of life

## Abstract

The objective of this study is to compare health-related quality of life (QOL) outcomes between radical prostatectomy (RP) and external beam radiation therapy (EBRT) for localized prostate cancer. PubMed, EMBASE, the Cochrane Library and Web of Science (to July 2017) were searched. Pooled analysis of each domain-specific score was calculated in relevant studies, and its change with follow-up time was explored by sub-group analysis. A total of six studies containing 4423 patients were included. Men underwent RP was associated with worse urinary and sexual domain score than EBRT (standardized mean difference (SMD) = –0.59, –0.58; 95% confidence interval (CI) = –0.73 to –0.45, –0.72 to –0.44). In contrast, EBRT group had lower bowel domain score than RP group (SMD = 0.42, 95% CI = 0.33 to 0.52). The sub-group analysis revealed the most severe urinary and sexual QOL in RP as well as bowel QOL in EBRT group all happened in the first month post operation. The different performance of two treatments in three QOL domains diminished afterwards. Health-related QOL should be considered comprehensively when planning follow-up for men after RP or EBRT for localized prostate cancer.

## INTRODUCTION

Localized prostate cancer is classified by European Association of Urology (EAU) 2016 as in men with stage T1/T2, Nx/N0 and M0 [1] who are usually offered radical prostatectomy (RP), external beam radiation therapy (EBRT) and other treatments such as active surveillance, observation and brachytherapy. However, the optimal treatment of clinically localized prostate cancer remains controversial.

Health-related quality of life (QOL) is an increasingly important end-point in localized prostate cancer which was mostly measured using the Expanded Prostate Cancer Index Composite Instrument (EPIC) [2, 3]. Randomized controlled trials (RCTs) [4–7], systematic reviews and meta-analysis [8–10] published so far have focused particularly on clinical oncological outcomes such as overall and cancer-specific survival, with little attention to patient-reported outcomes like health-related QOL. So we conduct this systematic literature review and meta-analysis of QOL outcomes reported by men after RP and EBRT for localized prostate cancer to compare the difference in the extent and duration of impaired QOL between the two treatments.

## RESULTS

### Included literature and related information

**Figure 1:**
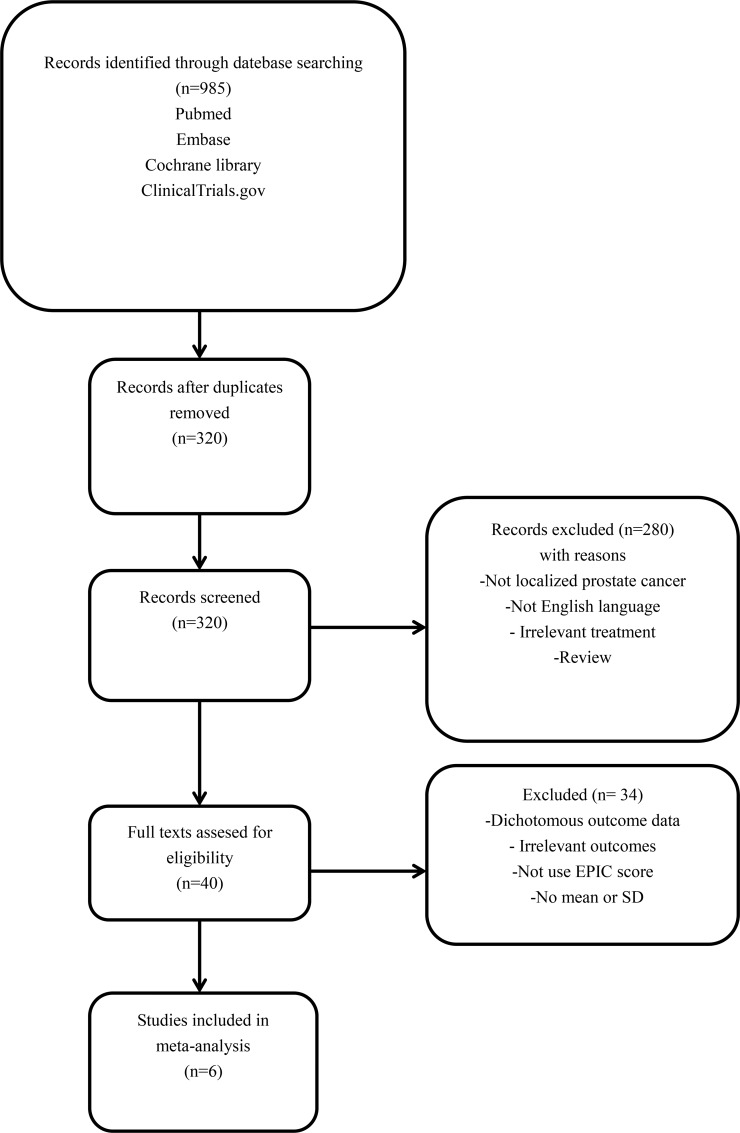
Flow chart illustrating the selection of studies for this meta-analysis

A total of 985 papers were obtained, and six of them were finally included according to the inclusion criteria [11–16]. See Figure 1 for the literature search process. Included literature covered a total of 4423 patients. Among them, 2615 men underwent RP and 1808 with EBRT. The follow-up time was one month to 15 years. The quality scores of the studies according to the Newcastle-Ottawa scale varied from 4 to 7, with a mean of 6. All studies were incorporated into the subsequent analysis. See Table 1 for literature information.

**Table 1:** Characteristics of studies included in this meta-analysis

Study	Design	QOL measure	Patient numbers	Treatment cohorts	QOL domain	NOS score	Follow-up time
Sanda 2008	Prospective study	EPIC	RP:603 EBRT:292	RP: Retropubic, laparoscopic or robot-assisted techniques with nerve-sparing at the surgeon's discretion EBRT: Intensity-modulated radiotherapy or highly conformal techniques with ADT	Sexual, Bowel QOL	7	2, 6, 12, 24 months
Katz 2012	Retrospective study	EPIC	RP:123 EBRT:216	RP: Retropubic prostatectomy with nerve-sparing at the surgeon's discretion EBRT: 35 Gy in the first 38 patients and 36.25 Gy in the remaining without ADT	Urinary, Sexual, Bowel QOL	5	1, 6, 12, 24, 36 months
Ferrer 2008	Prospective study	EPIC	RP:134 EBRT:205	RP: Retropubic prostatectomy with nerve-sparing at the surgeon's discretion EBRT: 3D conformal technique	Urinary, Sexual, Bowel QOL	6	3, 6, 12, 24 months
Donovan 2016	RCT	EPIC	RP:553 EBRT:545	RP: Open retropubic, nerve-sparing approach EBRT: 3D conformal radiotherapy at a total dose of 74 Gy with ADT	Urinary, Sexual, Bowel QOL	7	6, 12, 24, 36, 48, 60, 72 months
Resnick 2013	Prospective study	EPIC	RP:1164 EBRT:491	RP, EBRT	Urinary, Sexual, Bowel QOL	7	6, 12, 24, 60, 180 months
Nicolaisen 2014	Cross-sectional survey	EPIC	RP:38 EBRT:59	RP, EBRT	Urinary, Sexual, Bowel QOL	4	36 months

Abbreviations: RP, radical prostatectomy; EBRT, external beam radiation therapy; BT, brachytherapy; EPIC, Expanded Prostate Cancer Index Composite; QOL, quality of life; ADT, androgen deprivation therapy; NOS, Newcastle–Ottawa scale; RCT, randomized controlled trial.

### Urinary quality of life

**Figure 2:**
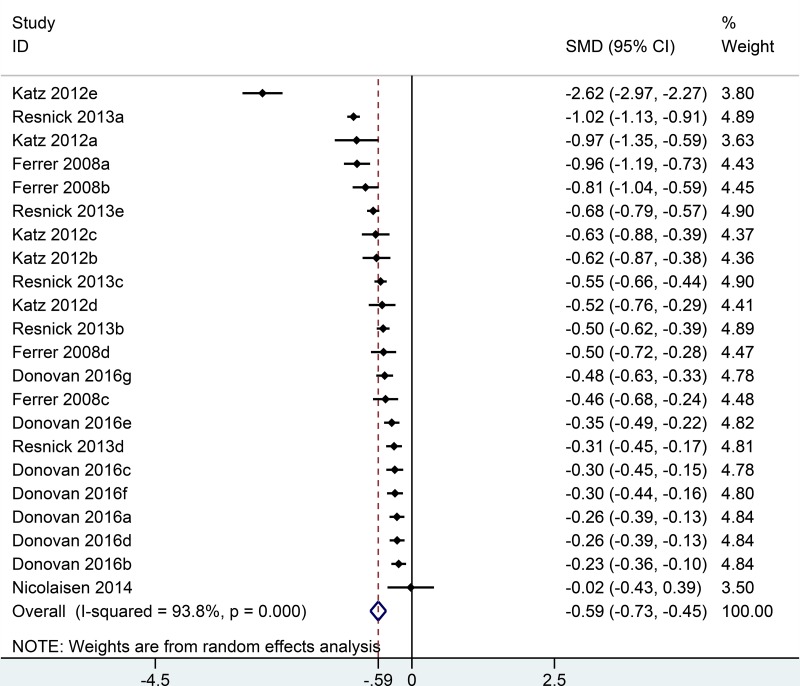
Forest plot showing urinary QOL of RP compared with EBRT

Five studies [12–16] reported urinary quality of life in the analysis, including 22 comparisons at different follow-up time (Figure 2). Generally, patients undergoing RP had lower urinary domain scores than men undergoing EBRT (SMD = –0.59; 95% CI = –0.73 to –0.45) (Supplementary Table 1). In sub-group analysis, compare to EBRT group, RP group had the lowest urinary domain scores in the first month (SMD = –2.62; 95% CI = –2.97 to –2.27) and experienced a sharp increase in the following two months (SMD = –0.81; 95% CI = –1.04 to –0.59) (Supplementary Table 1). The gap between RP and EBRT was narrowing over the years and only minimal difference existed in the 15th year (SMD = –0.31; 95% CI = –0.45 to –0.17) (Figure 5).

### Sexual quality of life

**Figure 3:**
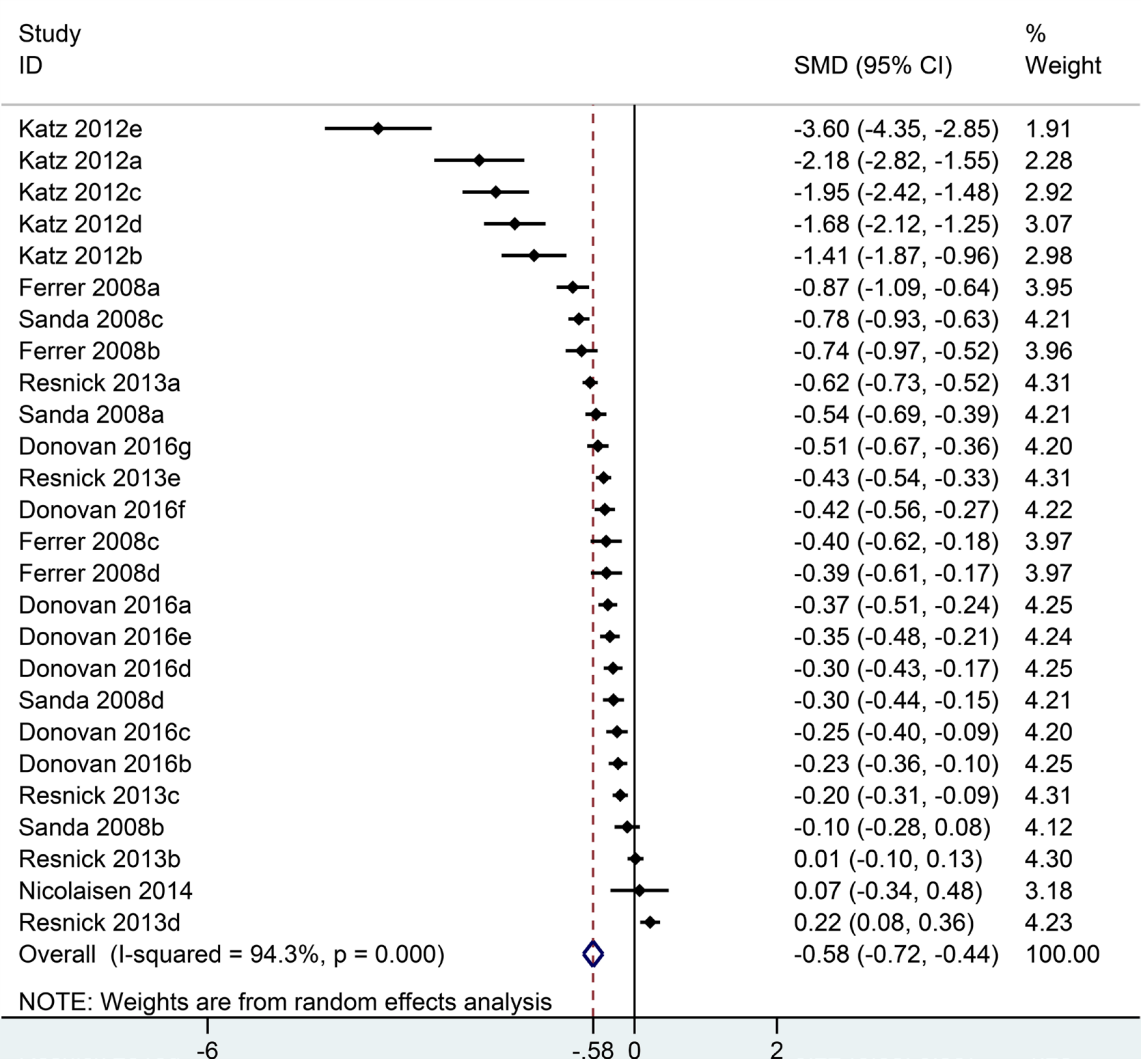
Forest plot showing sexual QOL of RP compared with EBRT

Six studies [11–16] reported sexual quality of life in the analysis, including 26 comparisons at different time points follow-up (Figure 3). Generally, patients undergoing RP had lower sexual domain scores than men undergoing EBRT (SMD = −0.58; 95% CI = –0.72 to –0.44) (Supplementary Table 1). In sub-group analysis, compare to EBRT group, RP group had the lowest sexual domain scores in the first month (SMD = –3.60; 95% CI = –4.35 to –2.85) and experienced a sharp increase in the second month (SMD = –0.78; 95% CI = –0.93 to –0.63). The gap between RP and EBRT was diminished afterwards and got to the minimum difference in the fifth year (SMD = –0.11; 95% CI = –0.35 to 0.14) (Supplementary Table 1). In the 15th year, sexual quality of life was slightly better for RP than EBRT group (SMD = 0.22; 95% CI = 0.08 to 0.36) (Figure 5).

### Bowel quality of life

**Figure 4:**
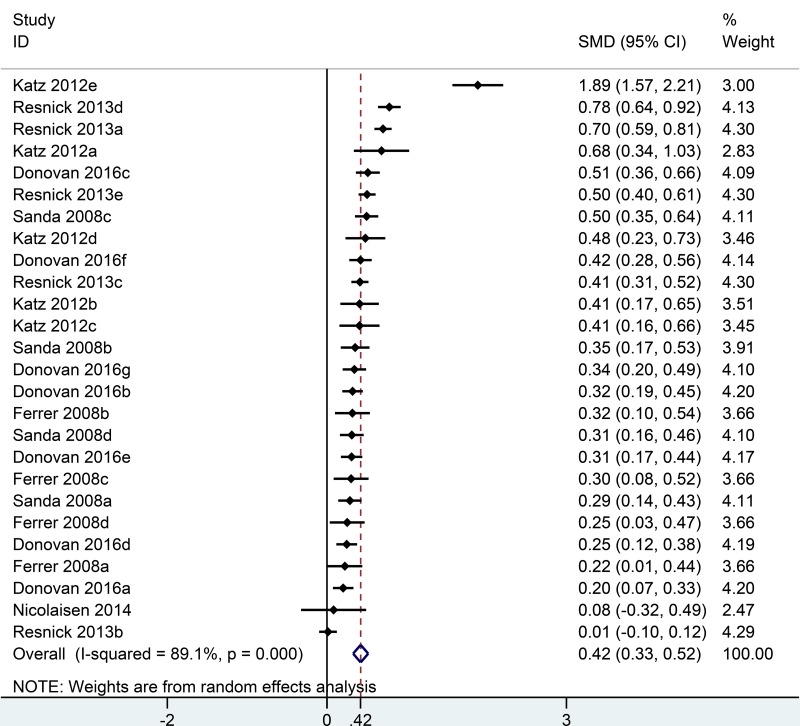
Forest plot showing bowel QOL of RP compared with EBRT

**Figure 5:**
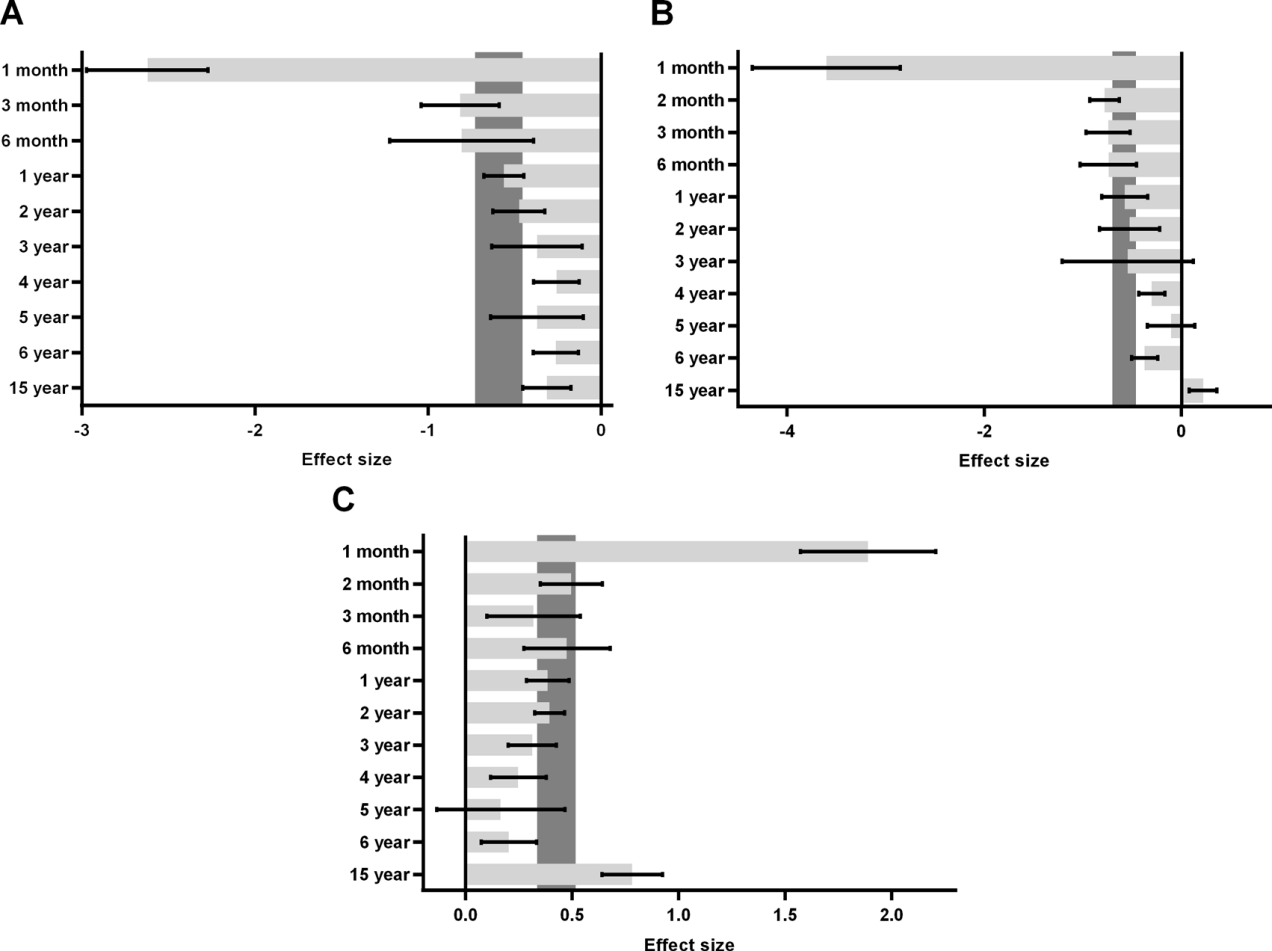
Sub-group analyses in urinary (**A**), sexual (**B**) and bowel (**C**) QOL stratified by follow-up time.

Six studies [11–16] all reported bowel quality of life in the analysis, including 26 comparisons at different time points follow-up (Figure 4). Generally, patients undergoing RP had higher bowel domain scores than men undergoing EBRT (SMD = 0.42, 95% CI = 0.33 to 0.52) (Supplementary Table 1). In sub-group analysis, compare to EBRT group, RP group had the highest bowel domain scores in the first month (SMD = 1.89; 95% CI = 1.57 to 2.21) and experienced a sharp decrease in the second month (SMD = 0.50; 95% CI = 0.35 to 0.64). The difference between RP and EBRT was shortening over the time and got to the minimum in the fifth year (SMD = 0.17; 95% CI = –0.14 to 0.47) (Supplementary Table 1). Afterwards, rebound happened in the sixth year (SMD = 0.20; 95% CI = 0.07 to 0.33) and reached to a new peak in the 15th year (SMD = 0.78; 95% CI = 0.64 to 0.92), indicating EBRT may have long-term bowel side effect (Figure 5).

## DISCUSSION

Current standard therapies for clinically localized prostate cancer include RP and EBRT. RP is the standard option to treat localized prostate cancer; the open retropubic and minimally invasive surgical technique are used the most widely. EBRT utilizes an external source of radiation to treat the prostate gland for men with localized prostate cancer. Cancer-specific outcomes between EBRT and RP have been proven comparable by randomized controlled trial and observational data [17–19].

Disease-specific health-related outcomes regarding QOL are other essential components of decision making for men with prostate cancer [20–22]. Two papers published back-to-back in JAMA this year highlighted this issue. One study [23] proved RP was associated with a greater decrease in sexual function and more urinary incontinence than EBRT. In another study [24], sexual function in men decreased more markedly with RP than EBRT. EBRT was associated with acute worsening of urinary obstructive and bowel symptoms. The differences declined with time in these symptoms and had largely disappeared by 2 years. In the ProtecT trial, patient-reported QOL parameters were assessed for urinary, sexual, and bowel function [14], which results were in line with the above studies, showing worse urinary symptoms and sexual function related to surgery, and more bowel symptoms related to RT. Large literature had reported both short- and long-term functional outcomes after RP or RT for localized prostate cancer [15], [25–27]. A previous systematic review tried to provide clarity about QOL outcomes in receipt of RP and RT, but failed to make a meta-analysis [3]. After reviewing the relevant literature published so far, we found many studies report these functional outcomes only graphically. So we utilized computer software to take them out. As a result, data from six studies reporting health-related outcomes were pooled to complete this analysis.

Damage to the urinary sphincter can result in urinary incontinence following RP, particularly stress incontinence. In a multi-institution analysis that included 603 patients who had undergone RP, 52% of patients reported urine leakage two months after surgery [11]. By 12 and 24 months, this had reduced to approximately 15%. Roughly 7% continued to experience urinary symptoms at two years. During EBRT, approximately one half patient experienced urinary symptom. Several studies reported lower urinary tract side effects developed immediately post-EBRT [15, 16, 28–30], which mostly resolve within two years of treatment. However, Fransson [31] conducted a 15-year follow-up study and found that men had long-term urinary side effects of EBRT, which were stress and urge incontinence. So how sustained improvement in urinary function can be observed continues to be uncertain. One longitudinal study suggested that there was not any further improvement in the continence rate for men undergoing RP [32]. By contrast, another study proved prostate cancer patients with RP continue to recover urinary function after 12 month [33]. Our meta-analysis pooling EPIC domain urinary summary score proved urinary problems in patients undergoing RP developed most severely in the first month, dropped fast in the next two months and resolve constantly in the following 15 years. Both RP and EBRT group reached a comparable outcome regarding urinary QOL in the long-term. However, for the limitation of included studies, we cannot further compare the urinary function, bother, incontinence, irritation or obstruction situation to draw more exact conclusion.

In patients treated with RP and EBRT, sexual dysfunction occurred in most patients at two months and persisted after two years [11]. Larger-scale studies reported a higher incidence of sexual dysfunction in men treated with RP compared to EBRT [28, 34, 35]. We pooled EPIC domain sexual summary score to find out men treated with RP experienced impaired sexual QOL immediately after surgery, which returned fast in the second month and improved over time. In the 15th year, sexual QOL was slightly worse in the EBRT group than RP, which may be caused by the use of adjuvant androgen-deprivation therapy [11]. For the same reason, sexual function or bother items cannot be made clear separately.

Bowel symptoms, primarily urgency and frequency, although rare after RP, were reported by 20% of patients treated with EBRT [11], which can be explained by the close proximity of prostate gland to the rectum, thus, the gastrointestinal tract getting the major toxicities of tissue irradiation. Most men treated with EBRT experienced short-term bowel dysfunction, mostly diarrhea, urgency, or abdominal or rectal pain. Acute symptoms usually resolve within two month [11]. Our analysis pooling EPIC domain bowel summary score also drew similar conclusion that EBRT group had the highest incidence of bowel side effects in the first month and resolve quickly within two month which can be controlled well in the subsequent five years. However, unlike other studies [16, 36], we found bowel symptoms deteriorated 5 years later especially in the 15th year, indicating EBRT may have long-term bowel side effect which cannot be ignored. Unfortunately, bowel function or bother problems cannot be ascertained further.

Some limitations exist in this analysis. First, significant heterogeneity was detected between studies of each domain which may be attributable to different study design, treatment and follow-up time. Second, only English papers were analyzed in our study, so the language bias may exist. Third, for the limited studies included, the funnel plot and publication bias were not presented and discussed. Forth, due to lacking sufficient data, other common treatment modalities for localized prostate cancer such as active surveillance, watchful waiting, and brachytherapy were not compared with RP and EBRT in this meta-analysis. Moreover, the financial burden between the two interventions for localized prostate cancer were not compared in this analysis, given increasing awareness on the cost influence in patients’ decision making when choosing cancer treatments [37]. Finally, for the different versions or components of patient-reported outcome measures used, many good studies cannot be include in this analysis and we are unable to compare EPIC sub-score of each specific domain as we mentioned above, specifically, function and bother items of urinary, sexual and bowel QOL.

In conclusion, men treated with RP experienced an acute worsen with respect to urinary and sexual QOL in the first two months post operation, which also happened in EBRT with bowel function. The two treatment groups continued to relieve in all functional outcomes to have similar health-related prognosis in the long-term follow-up. The future decision-making process must take into consideration of health-related outcomes during therapies for localized prostate cancer.

## MATERIALS AND METHODS

### Selection criteria for relevant literature

We conducted this analysis based on the Preferred Reporting Items for Systematic Reviews and Meta-Analyses (PRISMA) guidelines. Studies included met the criteria: (1) men diagnosed with localized prostate cancer. (2) treatment group is RP and EBRT. (2) outcome data were presented or can be calculated as mean and standard deviations (SD); (3) health-related QOL outcomes were presented as EPIC domain summary scores, specifically, urinary sum score, sexual sum score and bowel sum score. Each domain sub-score contained function and bother items. (4) the most recent or representative study of the same author or group was selected to include. Studies were excluded based on criteria: (1) no mean or SD values can be got; (2) not written in English; (3) not use EPIC score as QOL measurement tool.

### Literature search strategies and quality assessment

English literature published before July 2017 was systematic searched in PubMed, EMBASE, the Cochrane Library and Web of Science using keywords: “Prostatic Neoplasms”, “Patient Reported Outcome Measures”, “quality of life”, “sexual dysfunction”, “urinary incontinence”, “impotence”, “bowel dysfunction”, “prostatectomy”, “radical prostatectomy”, “radiotherapy” and “radiation therapy”. The Newcastle-Ottawa scale was used to evaluate the quality of the included studies. Higher score means better methodology of studies. Literature quality was assessed by both authors (Chen C and Chen Z) independently and disagreement was resolved through discussion.

### Data extraction

Data such as patient demography, QOL measure, treatment cohorts, domains and follow-up time was obtained by two authors (Chen C and Wang K) independently. Mean and SD were extracted directly from the article or estimated from graphics using the GetData Graph Dizitizer. SD was recalculated from standard errors or 95% confidence interval (CI) when needed. Missing information was requested by mail from the first author or the corresponding author.

### Statistical analysis

Stata 12.0 was used for statistical analysis and GraphPad Prism 6.0 was used to draw the bar chart in the sub-group analysis. Standardized mean difference (SMD) was used to compare continuous variables with the same domain. All results were described by 95% CI. Health-related QOL data are measured by EPIC in urinary, bowel, sexual domains in this analysis. A summary score constructed for each domain was collected, which range from 0 to 100 with high values indicate better functioning and quality of life. For comparison purposes, men treated with RP were set as the experimental group and men treated with EBRT were set as the control group. That is to say, an effect size < 0 reflects worse QOL in men with RP and an effect size > 0 reflects worse QOL in men with EBRT. Heterogeneity between studies was evaluated by Cochran's *Q* test and *I*^2^ test. Studies with *P* < 0.05, I^2^ > 50% were deemed heterogeneous, and was analysed by the random effect model. Sub-group analyses were conducted in urinary, bowel, sexual domains stratified by follow-up time points.

## SUPPLEMENTARY MATERIALS TABLE


