# Supplementary material for: Comparisons of health-related quality of life among surgery and radiotherapy for localized prostate cancer: a systematic review and meta-analysis
Source: Oncotarget. 2017 Oct 5;8(58):99057–65. doi: 10.18632/oncotarget.21519 (PMC5716791; doi:10.18632/oncotarget.21519)
Supplement: Supplementary file 1 [file oncotarget-08-99057-s001.pdf]

Comparisons of health-related quality of life among surgery and radiotherapy for localized prostate cancer: a systematic review and meta-analysis

SUPPLEMENTARY MATERIALS

Supplementary Table 1: Sub-group analysis of RP vs. EBRT stratified by follow-up time

	Number of comparisons	Std. Mean difference (IV, random, 95%CI)	χ2	df	P*
Urinary quality of life	22	–0.590 [–0.728, –0.452]	340.75	21	< 0.001
1 month	1	–2.620 [–2.972, –2.269]			
3 month	1	–0.814 [–1.040, –0.588]			
6 month	4	–0.807 [–1.221, –0.392]			
1 year	4	–0.562 [–0.678, –0.446]			
2 year	4	–0.475 [–0.626, –0.325]			
3 year	3	–0.371 [–0.632, –0.109]			
4 year	1	–0.259 [–0.390, –0.128]			
5 year	2	–0.370 [–0.637, –0.103]			
6 year	1	–0.262 [–0.393, –0.131]			
15 year	1	–0.313 [–0.452, –0.174]			
Sexual quality of life	26	–0.581 [–0.718, –0.444]	439.87	25	< 0.001
1 month	1	–3.603 [–4.353, –2.852]			
2 month	1	–0.781 [–0.930, –0.633]			
3 month		–0.743 [–0.968, –0.519]			
6 month	5	–0.741 [–1.026, –0.457]			
1 year	5	–0.577 [–0.810, –0.345]			
2 year	5	–0.526 [–0.832, –0.220]			
3 year	3	–0.545 [–1.210, 0.119]			
4 year	1	–0.302 [–0.434, –0.169]			
5 year	2	–0.106 [–0.347, 0.135]			
6 year	1	–0.374 [–0.506, –0.242]			
15 year	1	0.220 [0.081, 0.359]			
Bowel quality of life	26	0.422 [0.328, 0.517]	228.61	25	< 0.001
1 month	1	1.890 [1.571, 2.208]			
2 month	1	0.497 [0.351, 0.642]			
3 month	1	0.319 [0.100, 0.538]			
6 month	5	0.476 [0.274, 0.678]			
1 year	5	0.386 [0.286, 0.486]			
2 year	5	0.394 [0.324, 0.464]			
3 year	3	0.313 [0.201, 0.426]			
4 year	1	0.247 [0.117, 0.378]			
5 year	2	0.165 [–0.136, 0.467]			
6 year	1	0.203 [0.074, 0.332]			
15 year	1	0.782 [0.639, 0.924]			

Note: *P value for test for subgroup differences.
